# P-1083. Implementation of a Sustainable Process for Mupirocin Decolonization across Adult ICUs in an Urban Safety Net Hospital

**DOI:** 10.1093/ofid/ofaf695.1278

**Published:** 2026-01-11

**Authors:** Angela L Cramer, Mary R Bartkus, Alison L Nelson, Rebecca Rudel, Jacqueline Steiner, Patricia Green, Astride Barnard, Sherine Henry, Michelle Betances, Cassandra Pierre, Tamar F Barlam

**Affiliations:** Boston Medical Center, Boston University, Boston, MA; Boston Medical Center, Boston, MA; Boston Medical Center, Boston University, Boston, MA; Boston Medical Center, Boston University, Boston, MA; Boston Medical Center, Boston, MA; Boston Medical Center, Boston, MA; Boston Medical Center, Boston, MA; Boston Medical Center, Boston, MA; Boston Medical Center, Boston, MA; Boston Medical Center, Boston, MA; Boston Medical Center, Boston, MA

## Abstract

**Background:**

Our hospital observed a significant increase in hospital-acquired MRSA bacteremia during 2023 and 2024 with half of these cases occurring in the adult ICUs. The benefit of MRSA decolonization on infection rates and the efficacy of mupirocin as a decolonization agent are well-documented. We tested a set of implementation strategies to improve the mupirocin ordering rate in the adult ICUs. The goal of this implementation study was to determine an effective, sustainable strategy for universal nasal decolonization across ICUs.

**Figure:**
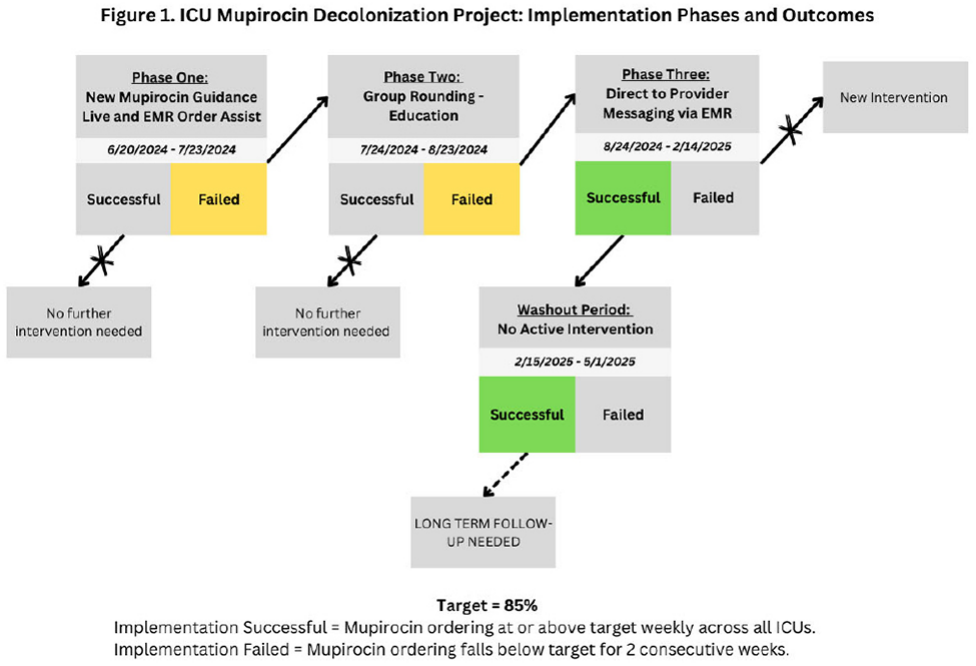

**Table 1.**
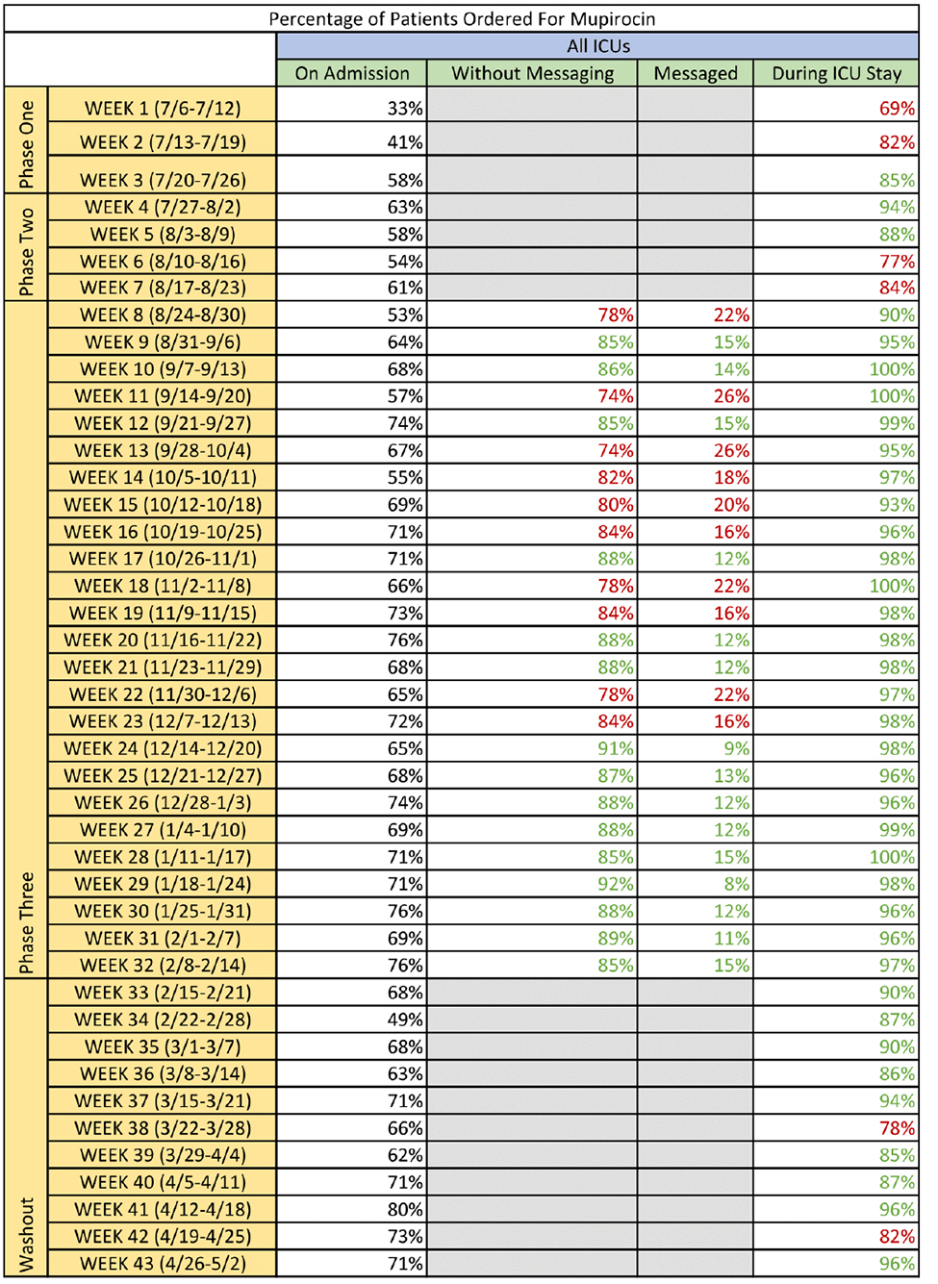
Percentage of Patients Ordered for Mupirocin across All ICUs Table shows how mupirocin was ordered across all adult ICUs during the three implementation phases and washout. Mupirocin was either ordered on admission via one of the admission order sets, ordered within 48 hours of the patient's ICU stay as an individual order, or not ordered. If the patient was not ordered for mupirocin within twelve hours of arrival to the ICU during phase three, the patient's first call provider received a direct message.

**Methods:**

There were three major implementation phases followed by a washout phase. The desired goal was ≥ 85% of patients ordered for mupirocin within 48 hours of admission. Two consecutive weeks below this goal prompted movement to the next phase.

Phase One

An email was sent to all ICU directors and chief residents announcing the new mupirocin decolonization initiative with all pertinent information including the go-live date. A FAQ document for nursing educators and nursing staff was created and disseminated.

Phase Two

Clinical staff were interviewed to identify areas for improvement and direct clinician education was provided.

Phase Three

Epic secure chat was used to directly message the first call provider of patients for whom mupirocin was not ordered per protocol.

Washout

A washout period was started after ordering rates were at goal for six consecutive months. This phase assessed the sustainability of the intervention without intensive auditing and feedback on each patient. Ordering trends were tracked, but providers were not directly messaged if mupirocin was not ordered for their patient within 48 hours.

**Table 2.**
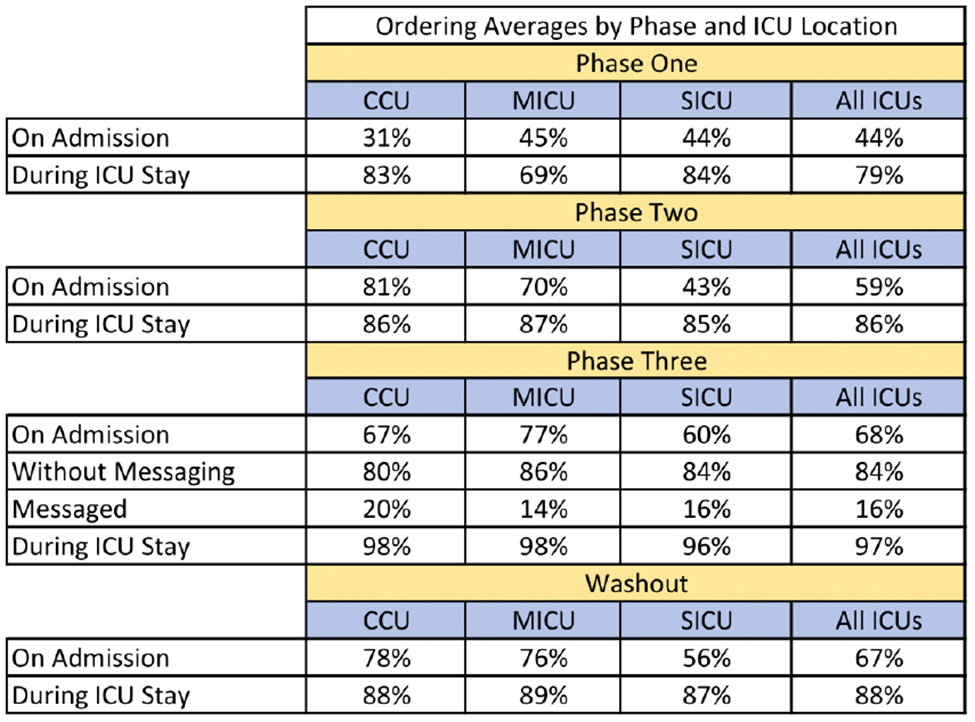
Ordering Averages by Phase and ICU Location

**Results:**

Across all ICUs, we did not reach the desired goal during the first two phases. After the first week of phase three, patients ordered for mupirocin during their ICU stay remained > 95% across all ICUs for the remainder of the phase. During the washout, there were only two, non-consecutive weeks where all ICUs were below 85%.

**Conclusion:**

The direct messaging to first call providers was the most effective strategy in maintaining mupirocin ordering for ≥ 85% of patients across ICUs. Ordering rates were maintained above goal during the washout. As such, further active interventions may not be necessary to sustain the target rates, but long-term follow-up is needed.

**Disclosures:**

All Authors: No reported disclosures

